# Dental Tumors

**Published:** 1868-12

**Authors:** Am. Forget

**Affiliations:** Member of the Imperial Society of Surgeons.


### ARTICLE II.

### *Dental Tumors.*

### FIBROUS ODONTOME.

By Am. Forget, Member of the Imperial Society of Surgeons.

(Translated from the French for the American Journal of Dental Science.)

To complete my notes on dental tumors, two varieties of which I have already indicated, first the eburnated odontome, and secondly the cementary odontome, there remains for study, a third variety, the fibrous odontome.

Already we have been able to see how, considering under the double point of view of histogeny and clinical history, the neoplasms developed in the thickness of the maxillary bones, we have been naturally led to establish between them original differences, which account for their diversity of structure, their difference of form, and the dissimilarity which, with their evolution, symptomatology and diagnosis, gives to each of them a characteristic physiognomy, which does not suffer them to be confounded under the vague, incorrect, and much too general name, of osteo-sarcoma, universally received to the present day, when the progress of anatomy and physiology no longer permits it to be retained.

Dupuytren has the honor of having first in France called the attention of surgeons to fibrous tumors of the jaws. Especially occupied with the clinical distinction between them and the morbid products springing from the organic structure of the bones themselves, this Surgeon devoted himself almost exclusively to bring into prominent notice the encysted character of these fibrous masses, without in any wise explaining himself, in his oral lessons, on their origin, structure and their point of departure.

Having had, during my prosectorship at Lisfranc's clinic, the opportunity of assisting in many operations for grave diseases of the jaw bone, I have dissected many specimens, and my researches on the composition and development of bony cysts, which I have described in my inaugural thesis, have authorized me to conclude that these fibrous products originate in the alveolar periosteum, of which they are usually only pathological processes. This histological opinion, which I have introduced into science, has made its own way in the profession, and is now generally adopted, but while it is justified anatomically in the majority of cases, and it almost always suffices to explain the mode of apparent development of the fibrous tumors under consideration, we must nevertheless acknowledge that certain neoplasms, with all the characters of those in the alveolar arch, and springing from over development of the dental bulb, cannot be referred exclusively to the same etiological influence; since they have a complex origin, as their anatomical constitution proves, when studied at an advanced period of their development.

This variety of fibrous tumor to which its composition and its origin give the name of dental fibroma or fibrous odontome, was unknown to pathologists when, in 1859, I brought forward the first examples of it, which I read to the Surgical Society, at its meeting on the 17th of August of the same year. I then said: "The knowledge of the anatomical elements originally existing under the laws of physiological development, and the analysis of the histological characters which differentiate the primordial tissues, by giving to each one of them its own physiognomy and special apti tudes, are the indispensible preliminaries to all serious studies in pathological anatomy. A partisan of the doctrine of naturalism which sees, in morbid products, only a departure from the mode of physiological growth, I have sought in my various labors on the diseases of the maxillary bones to establish this doctrine which now finds a new confirmation, in a case occurring under the following circumstances.

The case was that of a child 12 years old, presenting, apart from a tumor of the jaw, all the characters of health. The commencement of the disease dated from the age of 15 months, and was marked by a swelling of the right side of the face, and the formation of an abscess opening spontaneously on the inside of the mouth and on the cheek, where a fistula made its appearance. Taken to the hospital of

Nantes, he was examined by M. Letenneur, who ascertained the presence on the right side of the jaw, of a tumor raising the cheek and appearing to be as large as the fist. Examined from the mouth, it was found to occupy the whole of the right side of the jaw from the canine tooth which was comprised in it. This tumor is indented, covered by the mucous membrane, slightly ulcerated in a groove hollowed in its surface by the presence of the teeth of the upper jaw. Firm, elastic like a fibrous body, it is enclosed at its lower part in a very resisting bony shell. The canine, and the neighboring small molar are the only teeth seen on the tumor, being reversed, they are placed under the mucous membrane, which they have perforated.

The skilful surgeon of the Hotel Dieu at Nantes, took away this pathological product after having broken it up by tearing and cutting away the bony shell with Liston's shears. Of this he preserved only the base, which contained the dental vessels and nerves.

The cure completed at the end of six months, did not deceive our expectations. My learned colleague who considered the morbid product to be of a fibrous nature, and to be developed in the alveolo-dental periosteum, sent me the specimen with a letter, of which the following passage will seem on the one part to establish the nature of his diagnosis, and on the other part to demonstrate the originality of my personal researches, and the histological discoveries resulting from them :

"I have thought in transmitting this to your examination, Science might be the gainer by it, and I am sure it will give you pleasure, by allowing you again to verify the opinion expressed by you on the nature and origin of these tumours." To this kind appeal of the Hon. Mr. Letenneur, I replied by an anatomical description of the neoplasm which he had sent me and I have been led to bring forward a new variety of intra-maxillary fibroma which has never before been described.

*Anatomical description.* This tumor presented on its surface a raised series of mammillated protuberances separated from each other by depressions or flattened grooves between them. Besides, at numerous points the touch revealed a hardness and resistance belonging to bony tissue, which leaves no doubt concerning the complexity of the elements which compose it.

In truth covered and enveloped by a dense fibro cellular membrane of close texture and little vascularity, fixed thereby in a sort of aggregation which press them upon each other, its elements may decompose themselves into multiple tumors easy to isolate when the common envelope has been cut. Each tumor or lobe has an ovoid form and a variable size ; of its two extremities one is larger and turned towards the free surface of the neoplasm, e other smaller, answers to the base, where it becomes confounded with a very dense and resisting peripheral tissue, at that point where it constitutes the pedicle of the whole tumor, by means of which, the latter adheres to the maxillary bone.

Minute dissection of each of these lobes shows them to be disposed in regular order, and with constant symmetry of identical tissues. These tissues are, 1st, the external fibro-cellular tissue adhering quite feebly to the tissue which they cover, so that it is quite easy to remove it by a sort of peeling off; 2nd, of a tissue proper to each of the lobes, uniform and homogenious for all, whatever may be the difference in consistence and appearance. White, pearly and brilliant as a whole, a little yellow at some points, dry, firm and resisting, slightly elastic to pressure, moist and more friable over the lobules, this tissue appears to be of a fibrous nature. Between the proper tissues of these little tumors, and the external envelopes, there exists quite a number of plates and lamellae of bony tissue of a grayish yellow color, spongy, and offering but slight resistance to the edge of the knife.

Besides the details of organization which strongly characterize this fibrous product, among all the tumors which I had formerly observed, it should be stated that these teeth still existed in its substance, included in a cyst seeming to me to be formed of the alveolo-dental periosteum enormously developed ; one of these teeth corresponded on one of its sides to a deformed alveolus.

Independently of these three teeth, two bony tumors were found encysted, each in a distinct cell in the centre of this fibrous mass. These tumors resembling rather closely a truncated cone, are two alveoli, the ossified walls of which have a considerable thickness, as shown in a transverse section. Of these two alveoli, seregated in the mass of the morbid product, one contained a canine tooth partly thrust from its cavity by fibrous tissue in process of formation, which raised and pushed out the root of this tooth. The other alveolus contained only fibrous tissue, the fascicles of which were seen to emerge from the interstices of bony tissue Such a specimen, the histological interest of which is justified by its rarity, could not be lost to science. What was its intimate structure ? What prognosis could be deduced from it.

No doubt in considering this odd association of soft parts, of bony tissues, of encysted teeth, of alveoli isulated in the thickness of the morbid tissue, and especially the rapid evolution of the latter, this tumor might be regarded as the prototype of osteosarcoma of the jaw, at a period when the spirit of analysis applied to the study of each morbid tissue, had not yet revealed their distinctive characters. But since the fruitful researches which have illustrated the pathogeny of the maxillary bones, with the knowledge which we possess of the various anatomical elements, which originally concur in the constitution of the alveolo-dental organs, and which by their very complexity predispose the jaws to a series of lesions which cannot exist in the other parts of the skeleton; the determination of these, demanded a more severe study and a more rigorous diagnosis.

It results from the preceeding anatomical details, that this case offered a rare example of a hypertrophic transformation, or an excessive hypergenesis of the different elements, which enter into the composition of the alveolo-dental organs. It is a disease of dentition, since it is precisely at the period of the evolution of the teeth that the first morbid manifestations occur. In the space of two years ordinarily devoted to the physiological eruption of the molar teeth, and of the permanent canine, the tumor assumed a considerable development and advanced very rapidly. Recurring to the anatomical description and especially to the plate which reproduces the structural details, we are struck with the distinct, circumscribed form of these multiple tumors, which even at an advanced stage of their development, retain, so to speak, the impression of the alveolar mould which contained them at the beginning. The alveolo-dental periosteum, the different cellular fibrous elements of the odontogenic bulb, make an integral part of this neoplasm. Finally, the alveolus itself, as the hyperostosis of its walls shows, has participated in the excessive nutrition which has preceeded its development.

The following note which Mr. Chas. Robin has sent me, after having kindly studied, at my request, the microscopic characters of the morbid tissue, leaves no doubt as to its composition; showing that the tumor was one of the dental bulb and the alveolar periosteum, while still retaining the characteristic fundamental texture.

"What strikes us especially in the constitution of this tissue and which gives it a characteristic aspect, is the presence among the fibres which form its tissue, of a great number of elongated oval nuclei; these nuclei resemble the embryoplastic nuclei; many of these are a quarter or a half larger than are ordinarily found in the normal tissue. These nuclei, regarded from their extremity, have a spherical form, they are disposed quite regularly among the bundles of fibres of the cellular tissue which concur to form the morbid product.

Besides these elements we perceive fibro-plastic bodies of a star-like shape, resembling those which are observed near the surface of the dental bulb of young subjects and of the fœtus, and which of themselves form the enamel organ during the intra-follicular evolution of the tooth. It is known that these star-shaped fibro-plastic bodies instead of having two fusiform prolongations of greater or less length, furnish from two to five pale elongations upon the periphery of the central nucleus. Such was the case in this tumor and many of the prolongations of these fibro-plastic bodies had a considerable length. * * *

It was impossible not to recognize the analogy between the tissues of the tumor and that of the dental pulp in the fœtus.

The tissue of the laminated fibres is completely developed and only much more abundant in these tumors than in the normal organs. Besides it had a whiteish tint, dead to the naked eye, and a semi-opacity under the microscope, which the dental pulp does not possess. Besides, the vascularity of the morbid tissue was here manifestly less than in the normal pulp. However that may be, the comparative examination of the tissue of the latter and of the morbid tissue, shows that we have here undoubtedly a tumor derived from the dental bulb and preserving the characteristic fundamental texture of that organ. This texture is modified, it is true, by the superabundance of laminated fibres, by a difference in the properties of the different constituent elements, but without the intervention of other elements than those which enter into the composition of the dental bulb.

After this explanation, which shows the nature of the tissue in the numerous lobes and lobules which by their aggregation make up the whole of the neoplasm, without taking note of their multiplicity, we are naturally led to explain the latter by the original heterogeneousness of the bulbs themselves, of which each of these little tumors which have reproduced the histological composition, is in reality, only the normal or pathological expression.

In the various lobular tumors of which I have given a very exact drawing, we see under a form which varies with degree of development, and unequally distributed in each of the tissues which make them up, the bulb and alveolus which corresponds to it. Here it is the hypergenesis of the fibro-cellular, embryoplastic tissue which predominates ; the bony element abortive or absorbed, figures only in the state of laminæ and granulations, which disseminated in the periphery of the fibroid tumors, represent according to my views the rudimentary vestiges of the alveolus. Besides it is the excessive ossification of the alveolus which is the dominant character, so that the cavity is partly effaced by the considerable hypergenesis of its walls. It is an anatomico pathological fact, which had not before been indicated any more than the state of sequestration of the alveoli, completely isolated from the body of the jaw. These two points of morbid anatomy brought to notice by my labors, and the transformation or fibrous hypergenesis of the odontogenic bulb, equally shown by it confer upon them an incontestable originality and a very special interest.

I shall not insist upon the clinical precepts which a thorough and exact study of these tumors point out ; I have insisted upon them in my previous article, and shall confine myself to repeating that the homologies of these neoplasms, give them generally a benignant character, which allows us to extirpate them without necessarily removing with them the maxillary bone which serves as their receptacle. Let no one imagine, however, that the osseous cyst once opened, the fibroma can be easily and totally extracted by enucleation, as has been erroneously stated on the authority of a case of Dupuytren, which this skilful surgeon succeeded in relieving only after three successive operations. The enucleation, to be complete and instantaneous, would require not only that the tumor be encysted, but also that it should be free in the interior of the cyst, or that it should be attached only by superficial and limited connection. Now, observation has shown that intra-maxillary fibromas do not generally have this relation to the bony walls of their envelopes. They usually adhere by bundles of fibres which tie them firmly to the tissue of the bone especially at the base of the jaw, from which they can be separated only by many efforts to tear them away. It suffices to have made a single operation of this kind, and to have dissected a similar morbid product to ascertain the difficulties which must be overcome, and to know that it is very far from the operation necessary for a simple enucleation.

---

### ARTICLE III.

### *Notes from Dental Practice.*

